# 2166 Development of a statin risk communication tool for use in cancer survivors: A pilot

**DOI:** 10.1017/cts.2018.161

**Published:** 2018-11-21

**Authors:** Nirupa J. Raghunathan, Nassim Anderson, Emily Tonorezos, Deborah Korenstein

**Affiliations:** Memorial Sloan Kettering Cancer Center

## Abstract

OBJECTIVES/SPECIFIC AIMS: There are currently over a million survivors of childhood, adolescent, and young adult cancer in the United States, many of whom were treated with radiation therapy. Chest radiation with fields including the coronary arteries is a risk factor for cardiovascular disease. Of note, survivors are often unaware of this increased cardiovascular disease risk or, if they are aware, do not know how to mitigate the risk. Visual aids and communicating risk in terms of absolute risk reductions are shown to improve patients’ understanding. The Institute of Medicine recommends use of decision aids to optimize patient discussions of benefits and harms of therapies. Our goal is to develop and pilot test a statin therapy risk communication tool for use in high-risk cancer survivors to improve shared decision making and patient knowledge of coronary artery disease risk. METHODS/STUDY POPULATION: Participants were recruited from the adult long-term follow-up clinic at Sloan Kettering Cancer Center into 2 arms, usual care Versus intervention with the statin risk communication tool. The post-visit assessment used Likert-like scales to explore patient perceptions of statin use. The study was not powered for significance as it was a feasibility study; descriptive statistics were run to compare the 2 groups. RESULTS/ANTICIPATED RESULTS: Participants (n=45) had a mean age of 45. In the intervention group, 92% felt the information given was right compared with 73% of the usual care group. In all, 63% of the intervention arm felt the information was helpful, compared with 47% of those in usual care. And 53% of usual care would recommend the method to other patients and for other treatment choices compared to 67% of those in the intervention arm. DISCUSSION/SIGNIFICANCE OF IMPACT: This risk communication tool was assessed for acceptability and found to be more acceptable compared with usual care. In addition, we will gather further information on knowledge enhancement and decisional conflict as well as qualitative data regarding the shared decision making experience. With this information, a future randomized-controlled trial across institutions could provide information on how childhood, adolescent, and young adult survivors approach shared decision making with risk communication tools.

